# Descriptive study on the working conditions of residents of psychiatry in Madrid: working hours and 24-hour on call shifts

**DOI:** 10.1192/j.eurpsy.2022.291

**Published:** 2022-09-01

**Authors:** A. Cerame, P. Coucheiro, L. Cayuela, M. Maiques, A. Franco Soler

**Affiliations:** 1 Hospital Universitario José Germain, Hospital De Día, Leganes, Spain; 2 Hospital Universitario Severo Ochoa, Internal Medicine, Leganes, Spain; 3 Hospital Niño Jesús, Paediatrics, Madrid, Spain

**Keywords:** on call, resident physician, rest

## Abstract

**Introduction:**

Resident physician’s working conditions are linked to poor health outcomes of professionals and patient’s safety. Previous studies suggest that residents in Spain have difficulties enjoying mandatory rest after on-call shifts.

**Objectives:**

This study aims at describing the working conditions: working hours and the absence of mandatory rest periods after a 24h on-call shift in residents of psychiatry in the region of Madrid.

**Methods:**

A descriptive observational cross-sectional study was carried out through an anonymous survey adapted from the available literature.

**Results:**

Up to 24,1% of the surveyed residents could not enjoy mandatory resting periods after a 24 hour on-call shift and the mandatory weekly rest of at least 36 hours was not done in up to 17% of the cases with statistical significance (p <0.05). The average number of 24 hours on call shifts residents had to work per month was 5, which exceeds the maximum weekly hours allowed by law.

**Conclusions:**

The findings reveal a violation of resident physician labor rights in relation to resting times after on-call shifts, weekly breaks and working hours. These phenomena pose a significant threat to resident physician’s health and patient safety.

**Disclosure:**

No significant relationships.

